# Comment upon Dr. Wolff’s Case

**Published:** 1890-01

**Authors:** Geo. W. Sherbino

**Affiliations:** Abilene, Texas


### COMMENT UPON DR. WOLFF'S CASE.

EDITORS OF HOMŒOPATHIC PHYSICIAN:

Dr. Wolff's case cannot be covered by the simillimum of any remedy.

She has a complexity of symptoms that cannot be covered by one drug. I have had just such cases to treat. They can be cured by careful study and the application of the similar remedy.

If I were going to begin on a case like that I would take down the most prominent symptoms, the most peculiar ones, and those that have appeared most lately. The distressing ones should be relieved first, and I should let the rest alone for some future time; in this way these symptoms and the disease condition will be vanquished.

After each remedy has done all it can, after proper waiting, attack the next group of symptoms that are the most distressing, and so on to the end, till there is no more battle to fight.

I will enumerate some of the most prominent remedies:

Ars., Bell., Cal-c., Carbo-veg., Can., Caust., Lyc., Lach., Medorrhinum, Nat-m., Rhus-tox., Sepia, Silicea, Sanicula, and "others."

The watery discharge was caused by the local remedies, and "cannot" be accepted only as an irritant to the vaginal mucous membrane. GEO. W. SHERBINO.

ABILENE, TEXAS.

